# Quality of Life and Its Psychosocial Predictors among Patients with Disorders of Gut–Brain Interaction: A Comparison with Age- and Sex-Matched Controls

**DOI:** 10.3390/healthcare12070757

**Published:** 2024-03-30

**Authors:** Agostino Brugnera, Chiara Remondi, Antonino La Tona, Greta Nembrini, Gianluca Lo Coco, Angelo Compare, Alice Cardinali, Alessandra Scollato, Fabio Marchetti, Matteo Bonetti, Marie Graciella Pigozzi

**Affiliations:** 1 Department of Human and Social Sciences, University of Bergamo, 24129 Bergamo, Italy; 2 Department of Psychology, Sapienza University of Rome, 00185 Rome, Italy; chiara.remondi@uniroma1.it; 3 Department of Psychological Sciences, University of Palermo, 90133 Palermo, Italy; 4 Poliambulatorio Oberdan, 25158 Brescia, Italy

**Keywords:** disorders of gut–brain interaction, functional gastrointestinal disorders, quality of life, psychological predictors

## Abstract

The disorders of gut–brain interaction (DGBIs) are a heterogeneous group of chronic conditions that greatly reduce patients’ quality of life (QoL). To date, biopsychosocial factors (such as gastrointestinal symptoms, alexithymia, and interpersonal problems) are believed to contribute to the development and maintenance of DGBIs, but their role in affecting patients’ QoL is still under investigation. Out of 141 patients seeking treatment for their gastrointestinal symptoms, 71 were diagnosed with a DGBI (47 females, 66.2%; Mage: 41.49 ± 17.23 years) and were age- and sex-matched to 71 healthy controls (47 females, 66.2%; Mage: 40.45 ± 16.38 years) without any current gastrointestinal symptom or diagnosis. Participants completed a sociodemographic and clinical questionnaire and a survey investigating several psychosocial risk factors. We found greater symptom severity and difficulties in identifying feelings among patients compared to controls. Further, multiple linear regression analyses evidenced that, among patients, higher expressive suppression of emotions, difficulties in identifying feelings and interpersonal problems, and a lower cognitive reappraisal of emotions predicted lower QoL. Data suggest that the QoL of patients with DGBIs is affected not only by common risk factors (e.g., interpersonal problems) but also by specific difficulties in processing and regulating emotions. The implications of these findings are discussed.

## 1. Introduction

The disorders of gut–brain interaction (DGBIs), formerly known as functional gastrointestinal disorders (FGIDs), are complex, multidetermined conditions characterized by chronic or recurring gastrointestinal symptoms that are not explained by known structural or biochemical abnormalities [1,2,3]. Despite the overlapping symptoms and shared pathogenesis with a broad range of organic pathologies [4], DGBI diagnoses are made according to the Rome-IV criteria, which rely on clinical symptoms while excluding the presence of inflammatory, metabolic, or structural abnormalities in the gastrointestinal (GI) tract [1,2]. To date, 33 distinct adult DGBIs covering the whole spectrum of the GI tract can be diagnosed, the most common of which are irritable bowel syndrome (IBS), functional dyspepsia, and functional constipation [1,3]. DGBIs account for at least one-third of the referrals made to gastroenterology clinics [5] and affect approximately one-third of the world population [6]. For instance, a recent global survey involving more than 70,000 adults from 24 different countries evidenced that 40% of the entire sample met the criteria for at least one DGBI and these individuals had higher rates of healthcare utilization and a lower quality of life than those who did not meet criteria for a DGBI [6].

Quality of life (QoL) is a broad multidimensional concept with several dimensions, including lower levels of psychological distress, greater physical and social functioning, general mental and physical health, and vitality [7]. Several previous research studies, e.g., [8,9,10,11], and meta-analytical evidence [12,13] have shown that the chronicity of gastrointestinal symptoms, the lack of structural organic features, and healthcare-related costs greatly impact the QoL of patients with a DGBI. Moreover, when compared with healthy controls, previous research has shown that patients with a DGBI have significant impairment in both mental and physical components of QoL [3,13]. Despite the importance of QoL among patients with a chronic disease, the variables that explain reductions in QoL among those with DGBIs are still under-investigated. 

Considering the biopsychosocial pathophysiology of DGBIs [14], biological (e.g., gastrointestinal symptoms), psychological (e.g., anxiety and depression, emotion dysregulation, insecure attachment, alexithymia, somatosensory amplification), and social (e.g., interpersonal problems) factors may play an important role in the development and maintenance of poor QoL.

In regard to biological factors, patients with DGBIs experience chronic GI symptoms (e.g., abdominal pain, diarrhea, or constipation) that are—by definition—associated with an impaired health-related QoL [6,15]. For example, a large-scale multinational survey evidenced that these disorders impose a substantial burden on patients [6].

Regarding psychological factors, previous studies have suggested that psychological comorbidities significantly impact the QoL of patients with DGBIs, even more so than GI symptoms [16]. Hence, psychological factors interact with gastrointestinal symptoms and form a vicious circle that can slow recovery and exacerbate healthcare seeking [17]. Interestingly, psychiatric comorbidities are common among those with DGBIs, occurring in one-third up to half of diagnosed patients [14,18,19]. Some researchers highlighted the role of other psychological variables in affecting the QoL of patients with DGBIs, such as emotion regulation strategies (i.e., cognitive reappraisal and expression suppression) [20,21], insecure attachment (i.e., attachment anxiety and avoidance) [22,23], and alexithymia (i.e., difficulties in identifying and describing feelings) [24,25,26,27,28,29]. Moreover, somatosensory amplification (SSA, or the tendency to perceive somatic sensations as intense, bothersome, and/or harmful), has been found to be significantly higher among patients with DGBIs than controls [30].

Regarding social factors, previous studies have found that most patients with DGBIs have interpersonal relations characterized by non-assertiveness, submissiveness, and inhibition, which, if persistent, aggravate GI symptoms and QoL [31,32]. For instance, in a study on 235 patients with IBS, Lackner et al. [31] found that both low social support and negative interactions were associated with greater stress and a lower QoL, suggesting that the persistent pain and suffering experienced by patients require considerable support and guidance from others that is often perceived as difficult to meet.

Despite the growing number of studies on the psychosocial correlates of DGBIs [16,19], most of the previous literature is methodologically limited, being characterized by diagnoses made through self-report measures, unmatched control groups, or investigation of a small number of biopsychosocial correlates. Thus, the aims of this study were two-fold: (i) to test for significant differences between carefully selected patients with a DGBI and age-, sex-, and education-matched controls in several biological (e.g., gastrointestinal symptoms), psychological (e.g., anxiety and depression, emotion dysregulation, insecure attachment, alexithymia, somatosensory amplification) and social (e.g., interpersonal problems) factors; and (ii) to examine the potential biopsychosocial predictors of QoL, among both patients with DGBIs and healthy controls.

## 2. Materials and Methods

### 2.1. Procedure

From April 2021 to June 2022, all consecutive, treatment-seeking patients who self-referred (or were referred by their family physician) to a secondary center in northern Italy (Poliambulatorio Oberdan, Brescia, Italy) for their gastrointestinal problems were approached to take part in this study. Of those, 141 volunteered, signing informed consent forms and completing a paper-and-pencil survey while in the waiting room. In total, 34 individuals did not return for a follow-up (thus, their diagnosis was not confirmed) and 36 had GI problems due to a medical condition (e.g., ulcerative colitis, drug-induced liver damage, hepatocellular carcinoma), while the remaining 71 patients received a diagnosis of DGBI from an expert gastroenterologist according to Rome-IV criteria [2]. Of note, patients with DGBIs were diagnosed only after undergoing one or more medical exams to rule out any medical condition(s) that may have affected the onset/maintenance of their GI symptoms.

Once patients began to be enrolled in the study and thus their information started to be available to researchers, we recruited participants with similar sociodemographic characteristics (i.e., same sex, age [±5 years] and years of education [±5 years]) from the general population. A total of 264 individuals volunteered to fill out an online survey, 126 of whom were excluded due to self-reported gastrointestinal problems. Of the remaining 138 participants, 71 were age- and sex-matched to DGBI patients. Those who were excluded (i.e., 67 individuals) differed in one or more sociodemographics from DGBI patients; in those few cases where two or more participants were matchable with a specific patient, we randomly selected one through an online random list generator. These healthy controls (HC) declared to be free of a diagnosis of DGBI or GID and of current gastrointestinal symptoms. Healthy participants did not receive compensation and were recruited through a snowball procedure—starting from friends and relatives of the researchers—or self-referred responses to notices placed on social media platforms.

### 2.2. Participants

**Figure 1:**
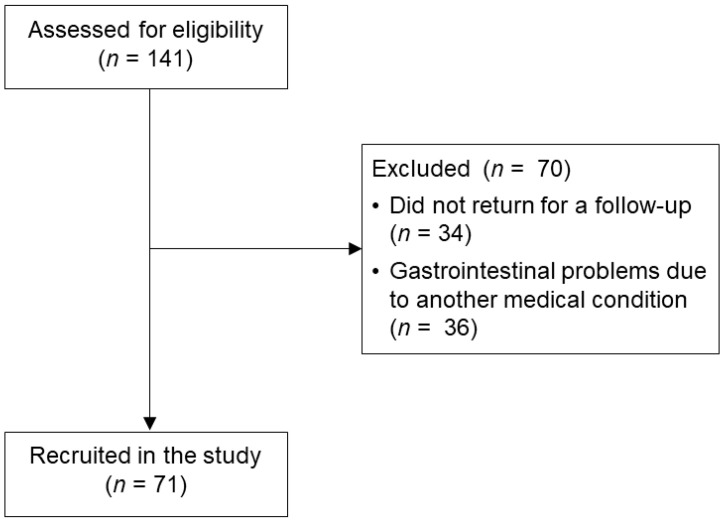
CONSORT diagram of the patients’ flow in the study.

A total of 71 patients with a diagnosis of DGBI (47 females, 66.2%; mean age: 41.49 ± 17.23 years; range 19–82 years) volunteered for this study. Patients attended high schools (*n* = 40; 56.3%) or university (*n* = 18; 25.4%), were mostly single, divorced, or widowed (*n* = 42; 59.2%), living with someone (*n* = 61; 85.9%), and workers (*n* = 48; 69.6%). Most patients underwent multiple visits for their GI problems (*n* = 40; 56.3%) and reported symptoms lasting more than one year (*n* = 47; 66.2%). The most prevalent diagnosis was IBS (*n* = 49; 69%). A flow diagram summarizing patients’ inclusion is provided in Figure 1.

We recruited an equal number of age- and sex-matched controls (47 females, 66.2%; mean age: 40.45 ± 16.38 years; range 18–83 years), whose sociodemographic characteristics have been described in Table 1. 

The inclusion criteria for patients were having a diagnosis of DGBI according to Rome-IV criteria [2], the absence of serious medical conditions (e.g., cancer, a diagnosis of any psychotic spectrum disorder), and of any physical condition that may have affected the GI symptoms. As for controls, the inclusion criteria were the absence of current GI symptoms, a diagnosis of either DGBI or any GI disorder, and any serious medical condition. Further, for both samples, an additional inclusion criterion was being a native Italian speaker. 

This study was approved by the Bio-Ethic Committee of the University of Bergamo and was conducted in accordance with ethical standards for the treatment of human experimental volunteers.

### 2.3. Measures

**Sociodemographic and clinical variables.** The sociodemographic data (e.g., age, sex, marital status) of all participants were collected through an ad hoc questionnaire, while the clinical data of DGBI patients were abstracted from their medical records or gathered during their gastroenterological visit.

**Quality of Life.** The Short Form Health Survey Questionnaire-6 (SF-36) [33,34] is a 36-item self-report multidimensional scale that evaluates both physical and mental components of health status. In the present study, we administered a total of 18 items, investigating general health perceptions, functioning, emotional role, mental health, and vitality (e.g., “*How much of the time during the past 4 weeks have you felt calm and peaceful?*”), which were summed into a total score. 

**Anxious and Depressive Symptoms.** The Hospital Anxiety Depression Scale (HADS) [35,36] is a 14-item self-report measure of psychological distress. The HADS is composed of two subscales, namely anxiety (7 items; e.g., “*I feel tense or ‘wound up’*”) and depression (7 items; e.g., “*I still enjoy the things I used to enjoy*”, reverse-item). 

**Gastrointestinal Symptoms.** The Gastrointestinal Symptoms Rating Scale (GSRS) [37,38] is a 15-item self-report measure of gastrointestinal symptoms (reflux, abdominal pain, indigestion, diarrhea, and constipation; e.g., “*Have you been bothered by pain or discomfort in your upper abdomen or the pit of your stomach during the past week?*”). 

**Emotion Regulation Strategies.** The Emotion Regulation Questionnaire (ERQ) [39,40] is a 10-item self-report measure of two commonly adopted emotion regulation strategies, namely cognitive reappraisal (5 items; e.g., “*I control my emotions by changing the way I think about the situation I’m in*”) and expression suppression (5 items; e.g., “*I keep my emotions to myself*”). 

**Somatosensory Amplification.** The Somatosensory Amplification Scale (SAS) [41,42] is a 10-item self-report instrument designed to assess the tendency to detect somatic and visceral sensations and experience them as unusually intense and alarming (e.g., “*I can sometimes hear my pulse or my heartbeat throbbing in my ear*”). 

**Attachment Dimensions.** The Experiences in Close Relationships-12 (ECR-12) [43,44] is a 12-item self-report measure of two dimensions of attachment to romantic partners, namely attachment avoidance (6 items; e.g., “*I want to get close to my partner‚ but I keep pulling back*”) and attachment anxiety (6 items; e.g., “*I need a lot of reassurance that I am loved by my partner*”). 

**Interpersonal Problems.** The Inventory of Interpersonal Problems-32 (IIP-32) [45,46] is a 32-item self-report measure of interpersonal problems and distress (e.g., “*I put other people’s needs before my own too much*”). The IIP-32 includes 8 subscales, which can be summed into a total score. 

**Alexithymia.** The Toronto Alexithymia Scale (TAS-20) [47,48] is a 20-item self-report measure of three dimensions of alexithymia, namely “difficulty describing feelings” (5 items; e.g., “*It is difficult for me to find the right words for my feelings*”), “difficulty identifying feelings” (7 items; e.g., “*I am often confused about what emotion I am feeling*”), and “externally-oriented thinking” (8 items; e.g., “*I prefer to analyze problems rather than just describe them*”). 

### 2.4. Statistical Analysis

Data were initially examined through simple descriptive statistics and zero-order Pearson correlations and tested for assumptions (i.e., internal consistencies of all scales, presence of univariate or multivariate outliers, and univariate normality). First, we reduced the number of main outcomes through a principal component analysis (PCA), entering as observed variables the HADS anxiety and depression scales and the SF-36 composite score. Assumptions, as well as the results of the PCA, were examined and interpreted according to guidelines [49]. Factor score coefficients (i.e., the estimated score of each participant on the underlying latent variable) were then computed through a “regression” method, which produces standardized scores with a mean of 0 and a variance of 1. The resulting new variable (i.e., the factor score) was used in subsequent analyses. 

We then tested for significant differences between patients with DGBIs and healthy controls on all psychological measures used in this study through independent sample *t*-tests. Finally, we examined the predictors of psychological QoL among patients with DGBIs and healthy controls through a multiple linear regression analysis, with all independent variables entered into the equation in one step (i.e., “enter” method). The dependent variable was the factor score obtained from the PCA, while the independent variables were the total scores of all psychological measures used in this study. 

Assumptions were checked before carrying out any statistical analysis [50], while missing data were treated as missing and not imputed. Analyses were performed with Statistical Package for the Social Sciences (SPSS) version 28.0.1. Effect sizes were computed and interpreted according to guidelines [51]. All statistical tests were two-tailed, and a *p* ≤ 0.05 was considered statistically significant.

## 3. Results

### 3.1. Between-Group Differences in Sociodemographic and Clinical Data

Sociodemographic and clinical data of all participants are reported in Table 1, while means and standard deviations for all psychological measures, separately for both groups, are reported in Table 2. 

Preliminary analyses evidenced that all scales had fair to good internal consistencies (see Table 3 for details). Variables were also normally distributed (i.e., kurtosis and skewness values were <|2| and |7|, respectively) [50]. We found no univariate or multivariate outliers, except for one participant from the HC group with heightened (i.e., >3 standard deviations) GSRS scores, which were brought into range [50]. 

No significant differences emerged between patients with DGBIs and HC on all sociodemographic variables (see Table 1).

### 3.2. Computing the Latent Variable “Quality of Life” (QoL)

As for the PCA on the different facets of QoL (i.e., HADS Anxiety and Depression, and SF-36 psychological QoL), the initial assumption checks were met (i.e., KMO = 0.704; Bartlett’s test of sphericity, *p* < 0.001). The PCA extracted a single factor (eigenvalue of 2.293, 76.44% of explained variance); all variables had excellent saturations with the latent component (SF-36, 0.908; HADS Depression, −0.879; HADS Anxiety, −0.835). The component, which we named QoL in accordance with previous literature [52], also showed a good internal consistency (α = 0.77). We then computed the latent factor score coefficients through a “regression” method, and used the resulting new variable (i.e., QoL) in subsequent analyses.

As for the Pearson correlations between psychological variables and QoL, in both samples we found that greater attachment anxiety, interpersonal problems, and difficulties in describing or identifying feelings worsened the psychological QoL. Interestingly, patients with DGBIs experienced impairments in QoL when they reported a greater somatosensory amplification and adopted the emotion regulation strategy “expressive suppression” to a greater extent (see Table 2). All these effects were small to large. 

Finally, among patients with DGBIs, QoL was unrelated to any sociodemographic and clinical variable (see Supplementary Table S1). 

### 3.3. Between-Group Differences in Psychological Variables (Aim 1)

We first tested for assumptions and found that few variables had non-homogenous variances (i.e., the Levene’s test was significant). When we compared both groups on all psychological variables tested in this study through independent sample Student or Welch’s (for variables with non-homogeneous variances) *t*-tests, we found that patients reported greater gastrointestinal symptoms and difficulties in identifying feelings than controls, with medium-to-large effects (see Table 3). 

### 3.4. Psychological Predictors of QoL among Patients with DGBIs and Healthy Controls (Aim 2)

We tested for this hypothesis through a multiple linear regression analysis. All assumptions were met (i.e., homoscedasticity, absence of multicollinearity, and the presence of a linear relationship between the dependent variable and the independent ones). 

The model was significant in both groups: DGBI, *F*(10, 52) = 4.822, *p* < 0.001, adjusted R^2^ = 0.381; HC, *F*(10, 60) = 7.921, *p* < 0.001, adjusted R^2^ = 0.497, with large effects. As shown in Table 4, greater interpersonal problems and difficulties in identifying feelings lowered the QoL among participants from both groups. As for the patient-specific results, QoL was significantly and positively predicted by cognitive reappraisal, and negatively predicted by expressive suppression. As for the control-specific results, another risk factor for a lower QoL was attachment anxiety. All the significant effects were medium to small. Sensitivity analyses—where we included as covariates both age and sex—led to the same results. 

## 4. Discussion

The aims of this study were (i) to test for significant differences between patients with DGBIs and matched controls in several biopsychosocial risk factors; and to examine the potential biopsychosocial predictors of QoL among both groups.

### 4.1. Between-Group Differences in Psychological Variables

In partial accordance with our first study aim, we found that patients with DGBIs significantly differed from healthy controls only in terms of greater GI symptoms and difficulties in identifying feelings. Other studies have reported similar findings [27,28,29,53]. For instance, Phillips et al. [53] found that “difficulties in identifying feelings” was one of the variables that most contributed to differentiating DGBI patients from controls. Similarly, Mazaheri et al. [29] found significant differences between these two groups in terms of alexithymia and the severity of GI symptoms. Recall that our sample was composed of treatment-seeking DGBI patients; one possible explanation of this finding is that symptom severity is one of the major factors causing patients to seek medical advice [54]. What matters most to patients with a chronic illness is the degree to which they are able to function and feel in their day-to-day lives. Therefore, whether their symptoms are mild or severe, treatment-seeking patients with DGBIs repeatedly experience unpredictable symptoms that negatively impact their day-to-day QoL [55], motivating them to seek treatment. Moreover, given the inability to identify and distinguish between feelings and bodily sensations [27,47], patients with DGBIs and concurrent alexithymia may amplify and misinterpret their somatic sensations, thus experiencing more severe somatic symptoms that exacerbate and\or maintain their GI disorders. Curiously, QoL did not significantly differ between the two groups, and this finding was not in accordance with previous literature [6,10,55]. Compared to other studies, the dependent variable of our analyses was a latent factor score that took into account both psychological distress (i.e., anxious and depressive symptoms) and health-related QoL, thus focusing on both ends of the dimension “quality of life” (rather than just one as in most of the previous literature). Further, the effect size was medium, suggesting that with a larger sample this difference may have been significant.

### 4.2. Psychological Predictors of QoL among Patients with DGBIs and Healthy Controls

As for our second study aim, we found that greater interpersonal problems and difficulties in identifying feelings worsened QoL in both patients and healthy controls. That is, in accordance with previous literature, the quality of interpersonal relationships highly impacted people’s well-being [56], probably due to its direct effect on the subjective feelings of loneliness [56], considered one of the major determinants of a low psychological QoL [54]. As for difficulties in identifying feelings, it is well known that alexithymia is positively related to a lower QoL, both in the general population [57] and in DGBI patients [58]. Some have argued that individuals who struggle to describe their affects may rely to a greater extent on maladaptive coping strategies to regulate their emotions and\or stressors, subsequently experiencing high psychological distress and low QoL [57].

Interestingly, the results from our second study aim also evidenced that patients with DGBIs experienced impairments in QoL when they reported greater somatosensory amplification and adopted to a greater extent the emotion regulation strategy of “expressive suppression” and to a lesser extent that of “cognitive reappraisal”. Emotion regulation is a multidimensional construct encompassing processes that influence which emotions are experienced and how they are expressed and acted out [40]. It is well known that emotion regulation strategies (i.e., cognitive reappraisal or expressive suppression) can significantly impact the well-being of individuals [40]. That is, cognitive reappraisal is related to optimism, higher self-esteem, more positive emotions, closer relationships, fewer depressive symptoms, and higher life satisfaction [40]. On the contrary, expressive suppression is linked to more negative emotions, feelings of inauthenticity, rumination, avoidance of close relationships, lower self-esteem, more depressive symptoms, and lower general well-being [40]. These emotional regulation strategies may mediate the association between DGBI symptoms and (dys)functional emotional responses [59], subsequently leading to a lower (as in the case of expressive suppression) or higher (as in the case of cognitive reappraisal) QoL. As for somatosensory amplification, our results were in accordance with previous—yet scarce—literature on this topic, e.g., [60]. SSA has been defined as “the amplification of various types of perceived threats to the integrity of the body” [61]: one could argue that patients with DGBIs with active functional GI symptoms may experience to a greater extent health anxiety and worries, which then diminishes their QoL. Moreover, GI symptoms did not affect the QoL of patients with DGBIs, and this result was surprising. Although previous literature has suggested that greater GI symptoms are one of the main causes of the lower QoL among patients, e.g., [6,15], our results suggest that psychological (e.g., alexithymia) and social (e.g., interpersonal problems) factors are of great importance for QoL in patients with DGBIs.

Finally, as for the HC-specific results, we found that attachment anxiety was a significant negative predictor of QoL, but only among controls. Although previous studies have suggested that patients with DGBIs report higher degrees of attachment anxiety than HC, e.g., [22], which in turn intensify their expressed distress and lower their QoL [62], it may be possible that when the effects of other variables (e.g., emotion regulation strategies) are taken into account, attachment anxiety becomes a less strong predictor of QoL in patients with DGBI. This non-significant prediction may also be the result of a suppressor phenomenon (see [63]), which occurs when the zero-order relationship between a predictor variable and an outcome is significant but becomes non-significant when included in a multivariate model. Prior to drawing firm conclusions regarding the influence of attachment anxiety on the QoL of patients with DGBI, further research is needed.

### 4.3. Limitations

This study comes with a number of limitations. First, we recruited a small Italian sample of treatment-seeking patients with a confirmed diagnosis of DGBI. Thus, our analyses may be underpowered, and our results may not generalize to other cultures or specific subgroups of patients (e.g., those with IBS). Larger, cross-cultural studies are needed to extend our findings. Second, we only collected self-report data, so the accuracy of individual reports cannot be guaranteed, despite all measures used in this study demonstrating adequate reliability. Finally, we only collected cross-sectional data, and this did not allow us to test for any causal inference or longitudinal interactions between study variables. As such, future studies should collect additional data points that are warranted.

### 4.4. Conclusions

In conclusion, of all the investigated biopsychosocial variables, only greater GI symptom severity and difficulties in identifying feelings discriminated patients with DGBIs from the control group, and, among patients, higher expressive suppression, lower cognitive reappraisal, difficulties in identifying feelings, and interpersonal problems predicted a lower QoL. Given that multicomponent therapeutic strategies are usually employed for DGBIs [64], the present findings have clinical relevance for both mental health practitioners and gastroenterologists. On the one hand, clinicians can tailor therapeutic strategies for patients with DGBIs by addressing emotional regulation difficulties and interpersonal challenges, alongside managing GI symptoms. Improving patients’ skills in coping with emotionally stressful situations may contribute to enhancing their overall QoL. On the other hand, gastroenterologists should adopt a holistic approach in their assessments, recognizing that the psychological and social aspects of patients’ experiences are integral components of their well-being. By considering the multifaceted nature of these disorders, clinicians and gastroenterologists can collaborate to enhance both the clinical outcomes and QoL of individuals affected by DGBIs.

## Figures and Tables

**Table 1:** Sociodemographic and clinical characteristics of patients with disorders of gut–brain interaction (DGBI; *n* = 71) and healthy controls (HC; *n* = 71).

	DGBI	HC		
Variable(s)	Frequency (%)	Frequency (%)	Test Statistic	*p*-Value
Age, *mean* (*SD*)	41.49 (17.23)	40.45 (16.38)	U = 2448.50	0.769
Sex, female	47 (66.2%)	47 (66.2%)	χ^2^(1) = 0	1
Education			χ^2^(2) = 4.616	0.099
*University or Ph.D.*	18 (25.4%)	30 (42.3%)		
*High Schools*	40 (53.3%)	32 (45.1%)		
*Middle or Primary Schools*	13 (18.3%)	9 (12.7%)		
Civil Status			χ^2^(3) = 0.179	0.981
*Widowed*	2 (2.8%)	2 (2.8%)		
*Divorced*	5 (7.0%)	6 (8.5%)		
*Married*	28 (39.4%)	26 (36.6%)		
*Single*	36 (50.7%)	37 (52.1%)		
Work Status			χ^2^(3) = 5.740	0.125
*Student*	9 (13.0%)	19 (26.8%)		
*Unemployed*	5 (7.2%)	6 (8.5%)		
*Part- or Full-Time Worker*	46 (66.7%)	42 (59.2%)		
*Retired*	9 (13.0%)	4 (5.6%)		
Living Status			χ^2^(3) = 4.830	0.185
*Alone*	10 (14.1%)	9 (12.7%)		
*With parents*	18 (25.4%)	16 (22.5%)		
*With spouse and/or children*	41 (57.7%)	37 (52.1%)		
*With others*	2 (2.8%)	9 (12.7%)		
Drinking alcohol	39 (56.5%) *	50 (70.4%)	χ^2^(1) = 2.920	0.087
Smoking	14 (20.3%) *	19 (26.8%)	χ^2^(1) = 0.813	0.367
DGBI Diagnosis				
*IBS-U* (*Unspecified*)	10 (14.1%)	/	/	/
*IBS-C* (*predominant Constipation*)	17 (23.9%)	/	/	/
*IBS-D* (*predominant Diarrhea*)	12 (16.9%)	/	/	/
*IBS-M* (*Mixed*)	10 (14.1%)	/	/	/
*Other DGBI* (*e.g., functional dyspepsia*)	22 (31.0%)	/	/	/
Long-term symptoms onset (>1 year)	47 (66.2%)	/	/	/
History of previous visits for GI symptoms	40 (56.3%)	/	/	/
Presence of any comorbidity	37 (61.7%) *	/	/	/
Currently under pharmacological treatment	28 (51.9%) *	/	/	/
Family history of a GI disease	6 (10.5%) *	/	/	/
Previous surgical treatment for GI problems	11 (18.6%) *	/	/	/

Note. U = Mann–Whitney U test; χ^2^ = Pearson’s chi square test. * = Percentage computed on valid percent (i.e., without considering missing data).

**Table 2:** Zero-order Pearson correlations between QoL and all psychological variables examined in this study, separately for patients with disorders of gut–brain interaction (DGBI; *n* = 71) and healthy controls (HC; *n* = 71).

		DGBI		HC
Variable(s)	*n*	Correlation Coefficient *r*	*n*	Correlation Coefficient *r*
GSRS	66	−0.079	71	−0.227
SSAS	68	−0.256 *	71	−0.199
ERQ Cognitive Reappraisal	68	0.188	71	0.152
ERQ Expressive Suppression	68	−0.416 **	71	−0.197
ECR-12 Anxiety	68	−0.302 *	71	−0.596 **
ECR-12 Avoidance	67	−0.181	71	−0.150
IIP-32	68	−0.529 **	71	−0.610 **
TAS-20 Difficulty Describing Feelings	68	−0.273 *	71	−0.399 **
TAS-20 Difficulty Identifying Feelings	66	−0.471 **	71	−0.540 **
TAS-20 Externally Oriented Thinking	66	−0.078	71	−0.285 *

Note. GSRS = Gastrointestinal Symptoms Rating Scale; SSAS = Somatosensory Amplification Scale; ERQ = Emotion Regulation Questionnaire; ECR-12 = Experiences in Close Relationships Scale-12; IIP-32 = Inventory of Interpersonal Problems-32 total score; TAS = Toronto Alexithymia Scale-20. * *p* < 0.05, ** *p* < 0.01.

**Table 3:** Means, standard deviations, and results of independent sample *t*-tests on all psychological variables examined in this study, among patients with disorders of gut–brain interaction (DGBI; *n* = 71) and healthy controls (HC; *n* = 71).

		DGBI		HC					
Variable(s)	*α*	*n*	Mean (SD)	*n*	Mean (SD)	*df*	*t*-Value	*p*-Value	*d*
QoL	0.77	68	−0.17 (1.00)	71	0.16 (0.99)	137	−1.936	0.055	−0.33
GSRS	0.82	69	30.96 (9.35)	71	23.48 (8.27)	138	4.734	<0.001	0.86
SSAS	0.61	71	14.06 (4.93)	71	13.15 (5.54)	140	1.116	0.266	0.19
ERQ Cognitive Reappraisal	0.87	71	27.91 (8.44)	71	27.87 (8.78)	140	0.023	0.981	0.004
ERQ Expressive Suppression	0.64	71	12.96 (4.54)	71	13.21 (5.79)	132.14 *	−0.338	0.736	−0.06
ECR-12 Anxiety	0.86	71	3.58 (1.46)	71	3.33 (1.82)	133.79 *	1.071	0.286	0.18
ECR-12 Avoidance	0.89	70	2.46 (1.14)	71	2.78 (1.49)	130.55 *	−1.364	0.175	−0.23
IIP-32	0.87	71	33.09 (14.13)	71	35.08 (16.84)	140	−0.731	0.466	−0.12
TAS-20 Difficulty Describing Feelings	0.72	71	12.10 (4.25)	71	12.42 (5.05)	136.09 *	−0.414	0.680	−0.07
TAS-20 Difficulty Identifying Feelings	0.79	69	17.19 (6.18)	71	14.96 (5.96)	138	2.174	0.031	0.37
TAS-20 Externally Oriented Thinking	0.62	69	17.41 (4.37)	71	18.20 (5.77)	130.30 *	−0.916	0.361	−0.15

Note. α = Cronbach’s alpha; *df* = degrees of freedom; *d* = Cohen’s d; GSRS = Gastrointestinal Symptoms Rating Scale; SSAS = Somatosensory Amplification Scale; ERQ = Emotion Regulation Questionnaire; ECR-12 = Experiences in Close Relationships Scale-12; IIP-32 = Inventory of Interpersonal Problems-32 total score; TAS = Toronto Alexithymia Scale-20. * = *df*s were corrected to account for non-homogeneous variances.

**Table 4:** Results of the multiple linear regression analysis on QoL, among patients with disorders of gut–brain interaction (DGBI; *n* = 63) and healthy controls (HC; *n* = 71).

	DGBI				HC			
Variable(s)	*Beta*	*t*-Value	*p*-Value	Partial *r*	*Beta*	*t*-Value	*p*-Value	Partial *r*
Constant		0.484	0.630			3.628	0.001	
GSRS	−0.040	−0.380	0.706	−0.053	0.057	0.559	0.578	0.072
SSAS	−0.043	−0.355	0.724	−0.049	−0.007	−0.066	0.947	−0.009
ERQ Cognitive Reappraisal	0.261	2.164	0.035	0.287	0.024	0.257	0.798	0.033
ERQ Expressive Suppression	−0.325	−2.588	0.012	−0.338	0.108	0.982	0.330	0.126
ECR-12 Anxiety	0.147	1.065	0.292	0.146	−0.305	−2.722	0.008	−0.332
ECR-12 Avoidance	0.189	1.418	0.162	0.193	−0.050	−0.469	0.641	−0.060
IIP-32	−0.384	−2.702	0.009	−0.351	−0.364	−3.297	0.002	−0.392
TAS-20 Difficulty Describing Feelings	0.090	0.618	0.539	0.085	−0.056	−0.448	0.656	−0.058
TAS-20 Difficulty Identifying Feelings	−0.391	−2.643	0.011	−0.344	−0.328	−2.547	0.013	−0.312
TAS-20 Externally Oriented Thinking	0.153	1.234	0.223	0.169	0.042	0.373	0.711	0.048

Note. GSRS = Gastrointestinal Symptoms Rating Scale; SSAS = Somatosensory Amplification Scale; ERQ = Emotion Regulation Questionnaire; ECR-12 = Experiences in Close Relationships scale-12; IIP-32 = Inventory of Interpersonal Problems-32; TAS = Toronto Alexithymia Scale-20.

## Data Availability

The data presented in this study are available on request from the corresponding author.

## Supplementary Materials

The following supporting information can be downloaded at: https://www.mdpi.com/article/10.3390/healthcare12070757/s1, Table S1: Zero-order correlations between the GSRS and SF-36 total scores and all sociodemographic and clinical variables, among patients with DGBIs (*n* = 71).
