# Supplementary material for: Quality of Life and Its Psychosocial Predictors among Patients with Disorders of Gut–Brain Interaction: A Comparison with Age- and Sex-Matched Controls
Source: Healthcare (Basel). 2024 Mar 30;12(7):757. doi: 10.3390/healthcare12070757 (PMC11011672; doi:10.3390/healthcare12070757)
Supplement: Supplementary file 1 [file healthcare-12-00757-s001.zip › healthcare-2824923-supplementary.pdf]

Supplementary Table S1

Zero-order correlations between QoL and all sociodemographic and clinical variables, separately for patients with DGBI (n = 71) and healthy controls (HC; n = 71).

	DGBI		HC	
**Variable(s)**	***n***	**Correlation coefficient *r***	***n***	**Correlation coefficient *r***
Age	68	.179	71	.275*
Gender, female	68	.012	71	-.033
Education, higher	68	.074	71	.076
Civil Status, married	68	.178	71	.225
Work Status, workers	68	-.022	71	.098
Living Status, living with someone	68	-.023	71	-.065
Drinking alcohol	66	-.026	71	.034
Smoking	66	.009	71	-.126
FGID Diagnosis	66	.035		
Long-term symptoms onset (> 1 year)	66	.167		
History of previous visits for GI symptoms	68	.047		
Presence of any comorbidity	58	.084		
Currently under pharmacological treatment	53	-.050		
Family history of a GI disease	55	.120	71	.035
Previous surgical treatment for GI problems	57	-.071		

Note. DGBI = Disorders of Gut-Brain Interaction; HC = Healthy Controls; GI = Gastrointestinal. * p <. 05
